# A Novel Herbal Medicine for Dyslipidemia: Assessments in Experimental Models

**DOI:** 10.1155/2021/5529744

**Published:** 2021-04-22

**Authors:** Pham Ba Tuyen, Truong Thi Huyen, Dinh Thi Thu Hang, Pham Thi Van Anh

**Affiliations:** ^1^Traditional Medicine Hospital, Ministry of Public Security, Hanoi, Vietnam; ^2^Hanoi Medical University, Hanoi, Vietnam

## Abstract

Dyslipidemia substantially contributes to the risk of cardiovascular diseases. The polyherbal formulation has been a traditional therapeutic strategy used to treat dyslipidemia. This study was designed to evaluate the effects of a novel herbal medicine called “GANMO” on an experimental animal model with endogenous dyslipidemia and exogenous dyslipidemia. In the endogenous hyperlipidemia model, rats were previously treated with GANMO tablets and intraperitoneally injected with poloxamer 407 to induce hyperlipidemia. In the exogenous hyperlipidemia model, rats were given oral administration of oil-cholesterol mixture and GANMO for 4 consecutive weeks. Serum lipid profiles were assessed at all experimental animals. In both models, GANMO at both doses significantly decreased the serum total cholesterol (TC) level and non-high-density lipoprotein (HDL) cholesterol level as compared with the model group. HDL cholesterol levels increased in rats with high doses of GANMO compared to those with low doses. GANMO at both doses substantially reduced TG level in the endogenous hyperlipidemia model. In conclusion, GANMO tablets posed a positive effect on serum lipid modulations in dyslipidemia models.

## 1. Introduction

Dyslipidemia refers to the excess status of fatty substances including cholesterol, triglyceride, and decreased high-density lipoprotein cholesterol (HDL-C) in the bloodstream [1]. Besides, dyslipidemia is a major contribution to the onset of cardiovascular diseases (CVD) such as atherosclerosis, myocardial infarction, and cerebral vascular accidents, which are the main causes of the global burden of diseases [2]. Currently, although statins (e.g., simvastatin, atorvastatin, and rosuvastatin) have been widely used to reduce plasma lipids, their usage may be limited due to their side effects such as hepatotoxicity, rhabdomyolysis, or skeletal muscle injury [3]. Thus, natural alternatives have been proposed to control lipid metabolism.

Using multiple herbs (i.e., polyherbal) has been used in the traditional medicine approach given that it can improve the effects of therapy in curing a specific disease [4]. Several previous studies in Asian countries such as Indonesia [5] or China [6] have attempted to produce polyherbal medicine to treat dyslipidemia. In Vietnam, we have produced a novel polyherbal medicine called “GANMO” in improving serum lipid profiles. Each drug tablet contains *Pericarpium Citri Reticulatae*, *Atractylodes macrocephala Koidz.*, *Reynoutria japonica Houtt.*, *Folium Nelumbinis*, *Sargassum pallidum* (*Tum*), *Cassia tora L.*, *Alisma plantago aquatica L.*, *Fallopia multiflora* (*Thunb.*) Haraldson, *Crataegus cuneara Sied. et Zucc.*, and *Rheum palmatum L.* Several previous studies have shown the benefits of these herbal medicines in reducing serum lipid levels [7–9]. This study aimed to assess the effects of GANMO tablets on serum lipid profiles of dyslipidemia experimental animals.

## 2. Materials and Methods

### 2.1. Preparation of Materials

GANMO was prepared and offered in form of tablets, which contained *Pericarpium Citri Reticulatae* 0.83 g, *Atractylodes macrocephala Koidz.* 1 g, *Reynoutria japonica Houtt.* 1.25 g, *Folium Nelumbinis* 1.25 g, *Sargassum pallidum* (*Tum*) 1.25 g, *Cassia tora L.* 1.25 g, *Alisma plantago aquatica L.* 1 g, *Fallopia multiflora* (*Thunb.*) *Haraldson* 1 g, *Crataegus cuneara Sied. et Zucc.* 0.83 g, and *Rheum palmatum L.* 0.83 g. The tables were produced in the Traditional Medicine Hospital, Ministry of Public Security, Hanoi, Vietnam. The recommended dosage was 12 tablets per day.

The materials were prepared in compliance with the standards of Vietnamese Pharmacopoeia V. Grinding and filtering material procedures were implemented under the microbiological safety and Pharmacopoeia V requirements. Peels of *Pericarpium Citri Reticulatae*, roots of *Atractylodes macrocephala Koidz.*, fruits of *Reynoutria japonica Houtt.*, leaves of *Folium Nelumbinis*, *Sargassum pallidum* (*Tum*), seeds of *Cassia tora L.*, roots of *Alisma plantago aquatica L.*, roots of *Fallopia multiflora* (*Thunb.*) Haraldson, fruits of *Crataegus cuneara Sied. et Zucc*., and roots of *Rheum palmatum L.* were washed, removed impurities, and cooked twice. At the first time, materials were cooked for 8 hours from boiling, collecting the extract (I), and adding water. At the second time, materials were cooked for 2 hours from boiling and collecting the extract (II). Extracts (I) and (II) were put together and concentrated at temperature 60°C.

### 2.2. Animal Preparation

Wistar rats (males and females, weighing 180–220 g) were provided by the Center of Experimental Animals, DanPhuong, Hanoi, Vietnam. Healthy Swiss albino mice (of both sexes, weighing 21–25 g) were originated from the National Institute of Hygiene and Epidemiology (NIHE), Vietnam. Animals were housed under the standard environment (temperature: 25°C ± 2°C and relative humidity: 80% ± 10%), 12-hour dark/light time (lights on at 6:00 AM). We fed the mice with standard animal feed and allowed free access to water. After randomizing the rats into different intervention groups as well as before implementing the experiment, mice were transferred to the laboratory conditions for 7 days at the laboratory of Pharmacology Department, Hanoi Medical University. All protocols used in this study were approved by the Scientific Board Committee of Hanoi Medical University (ref. number: IRB00003121).

### 2.3. Chemical Preparation

In this study, we used propylthiouracil (Rieserstat®) 50 mg, cholesterol (Merk, Germany), cholic acid, poloxamer 407, (Sigma, Singapore), atorvastatin 10 mg (Stellapharm J.V. Co., Ltd.), and peanut oil (Vietnam). Biochemical analyzer (ERBA Chem, India) and commercial ERBA diagnostic kits were used for serum analysis of total cholesterol (TC), triglyceride (TG), and high-density lipoprotein-cholesterol (HDL-C).

### 2.4. Procedure

Exogenous dyslipidemia model: dyslipidemia was induced in rats by oral administration of cholesterol mixture (cholesterol 10%, cholic acid 1%, propylthiouracil (PTU) 0.5%, and peanut oil added to precisely 1 mL) for four weeks [10]. Wistar Albino rats (180–220 g) were divided into 5 groups with 10 rats per group. Rats were given oral medication twice per day, at least two hours apart:

  Group 1 (control group): distilled water 10 mL/kg b.w twice per day
  Group 2 (model group): cholesterol mixture 10 mL/kg b.w per day and distilled water 1 mL/100 g b.w per day
  Group 3 (atorvastatin): cholesterol mixture 10 mL/kg b.w per day and atorvastatin with the dose of 10 mg/kg b.w per day
  Group 4 (GANMO—low dose): cholesterol mixture 10 mL/kg b.w per day and GANMO at the dose of 15.1 g/kg b.w per day (equivalent to clinical dose)
  Group 5 (GANMO—high dose): cholesterol mixture 10 mL/kg b.w per day and GANMO at the dose of 45.3 g/kg b.w per day (three times as high as clinical dose)

Rat's body weight was recorded at baseline, after 2 weeks, and after 4 weeks. On day 15 and day 29, rats fasted overnight. Blood was collected to measure serum TC, TG, and HDL-concentrations. LDL-C concentration was calculated using the Friedewald formula: LDL-C = TC−(HDL-C)−(TG/2.2) (mmol/L) [11].

Endogenous dyslipidemia model: poloxamer 407- (P-407-) induced dyslipidemia model was described by Millar et al. [12]. Our mice were randomly separated into five groups with 10 rats per group.

  Group 1 (control group): mice were given oral distilled water 1 mL/100 g b.w/day and then received intraperitoneal (IP) injection 0.9% NaCl 10 mL/kg b.w on day 7.
  Group 2 (model group): mice were given oral distilled water 1 mL/100 g b.w/day and then received IP injection 2% P-407 at the dose of 200 mg/kg b.w on day 7
  Group 3 (atorvastatin): mice were given oral atorvastatin at the dose of 100 mg/kg b.w/day and then received IP injection 2% P-407 at the dose of 200 mg/kg b.w on day 7
  Group 4 (GANMO—low dose): mice were given oral GANMO at the dose of 30.2 g/kg b.w/day (equivalent to clinical dose); then received IP injection 2% P-407 at the dose of 200 mg/kg b.w on day 7.
  Group 5 (GANMO—high dose): mice were given per oral GANMO at the dose of 90.6 g/kg b.w/day (3 times as high as clinical dose) and then injected i.p. 2% P-407at the dose of 200 mg/kg b.w on day 7.

Blood was collected at 24 h after IP injection of P-407 and analyzed for serum lipids including TG, TC, and HDL-C. Non-HDL-cholesterol (non-HDL-C) was estimated: non-HDL-C = TC−(HDL-C).

### 2.5. Statistical Analysis

Data were analyzed using SPSS software version 20.0. *T*-test and ANOVA tests were performed to examine the differences in serum lipid profiles in different groups. A *p* value <0.05 was considered to be statistically significant.

## 3. Results

### 3.1. Effects of GANMO Tablets on Lipid Levels in Exogenous Dyslipidemia Model

Table 1 shows that, for all time points, no significant difference in body weight was found among groups (*p* > 0.05).

Table 2 shows that atorvastatin tended to decreased levels of TG, TC, and LDL-C, and GANMO at the dose of 45.3 g/kg had a tendency to reduce TG level as compared with the model group after 2 weeks of treatment, but no significant change was observed (*p* > 0.05).

**Figure 1:**
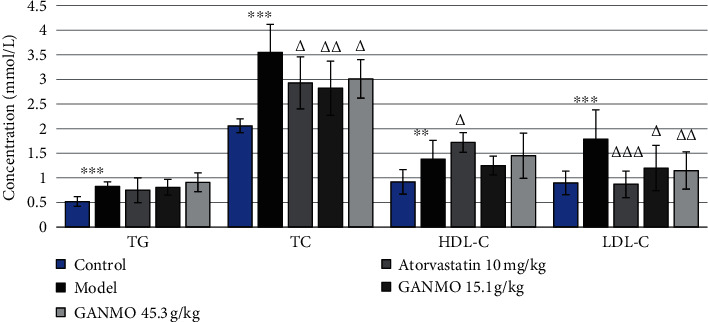
Changes in the serum lipid concentration of animals in cholesterol-induced dyslipidemia after 4 weeks.

Figure 1 and Table 3 show that, after 4 weeks of treatment, in the groups treated with atorvastatin and GANMO, there was a considerable decrease in levels of LDL-C and TC as compared with the model group. In terms of the HDL-C level, the group treated with GANMO at the dose of 45.3 g/kg tended to increase the HDL-C level, but no significant difference was observed (*p* > 0.05). No differential change was observed between groups treated with GANMO at the dose of 15.1 g/kg and the model group (*p* > 0.05).

### 3.2. Effects of GANMO on Lipid Levels in Poloxamer 407-Induced Dyslipidemia

Table 4 illustrated that there was a dramatic development in all lipid levels in the model group as compared with the control group (*p* < 0.001).

Table 5 shows that there was a substantial reduction in the TC level and non-HDL-C level and a significant increase in HDL-C level in the group treated atorvastatin as compared with the model group. GANMO at both doses of 90.6 g/kg b.w and 30.2 g/kg b.w significantly decreases TG level, TC level, and non-HDL-C level developing the HDL-C level as compared with the model group.

## 4. Discussion

In literature, the model of exogenous dyslipidemia induced by oil-cholesterol mixture (cholesterol with bile acid and hypothyroidism-inducing agents) is widely used to screen natural or synthetic drugs [13]. In this study, we evaluated the effects of GANMO tablets on the changes in serum lipid levels.

The findings of this study suggested that there was no significant difference in body weight among intervention and control groups at all time points after four weeks of the high-fat diet. However, the model of hyperlipidemia was induced successfully through the significant change of serum biochemical indexes. In particular, rats in the model group given the cholesterol-rich diet exhibited high plasma lipid levels, including TC, TG, and LDL-C levels. There was a significant improvement in serum lipid indexes in the group treated with GANMO tablets as compared with the control group after 4 weeks. The lipid-lowering effect of GANMO was a dose-dependent effect, and GANMO at a high dose posed a greater effect than the low dose.

A model of endogenous hyperlipidemia was developed by IP injection of P-407 200 mg/kg b.w. P-407, a polyether-based nonionic surface-active-agent (surfactant), providing an attractive means of inducing hyperlipidemia because of its rapid onset and seeming lack of over toxicity as compared with Triton WR-1339. P-407 has been known to cause significant dose-dependent hypercholesterolemia and hypertriglyceridemia in rats via several mechanisms (e.g., inhibition of lipoprotein lipase, indirect stimulation of HMG-CoA (3-hydroxy-3-methylglutarylCo-A) reductase, and promotion of concentration of hepatic cholesterol content) [14]. Based on its mechanism and shreds of evidence of efficiency, the statin is chosen as the drug reference standard. It inhibits HMG-CoA reductase, which counters the effect of P-407, thus decreases the serum TC. In addition, it also lowers the level of LDL by lowering the level of its precursor (VLDL and IDL), which further enhances its lipid-lowering effect. Currently, seven different statins are available generically, but only atorvastatin and rosuvastatin are used in high-intensity therapy [3].

Because of its safety, various P-407-induced hyperlipidemia models in rats and mice have been often conducted with the standard dose of 500 mg/kg. In this study, the dose of 200 mg/kg was chosen to evaluate and compare the effects of the regimens. Within 24 h of its IP injection, a profound hyperlipidemia state was achieved. Our finding showed that TG concentration increased substantially by 6.8-fold, and TC levels and non-HDL-C concentrations increased by 2.9-fold and 4.1-fold, respectively. Based on the success of the P-407-induced hyperlipidemia model, the effects of the GANMO tablet could be evaluated precisely. The data indicated that there was a substantial decrease in the levels of TG, TC, and non-HDL-C and a significant increase in the HDL-C level in dyslipidemia mice treated with GANMO at both doses of 30.2 g/kg b.w and 90.6 g/kg b.w. Mechanisms of compounds in GANMO tablets in the treatment are very complex.

These effects of GANMO tablets suggested the combination of lipid-lowering effects of herbal medicines. Yu et al. [7] suggested that polymethoxyflavonoids (PMFs) which were isolated from *Pericarpium Citri Reticulatae* (PCR) could be used to reduce serum lipid levels [7]. Meanwhile, in *Reynoutria japonica* Houtt, there are more than 70 compounds (such as quinones, stilbenes, or flavonoids) isolated and identified. These compounds were illustrated to regulate lipid metabolism and the effect on hyperlipidemia [8]. Flavonoids (epicatechin, myricetin, hyperoxide, quercitrin, and quercetin) were extracted from *Rheum palmatum L*. [14]. These flavonoids posed lipid-lowering effects through various research studies [15–18].

## 5. Conclusion

In conclusion, our results demonstrated that oral administration of GANMO at doses of 30.2 g/kg and 90.6 g/kg for consecutive 7 days significantly reduced levels of TC, TG, non-HDL-C and increased the HDL-C level in P-407-induced hyperlipidemic mice. The exogenous dyslipidemia model induced by oil-cholesterol mixture and oral administration of GANMO tablets for 4 weeks at both doses of 15.1 g/kg and 45.3 g/kg reduced TC level and non-HDL-C level in rats. Moreover, GANMO tablets at the dose of 45.3 g/kg tended to increase HDL-C level as compared with the model group.

## Figures and Tables

**Table 1:** Effect of GANMO tablets on body weight gain in dyslipidemia.

Groups	*n*	Initial weight ($\bar{X}$ ± SD)	After 2 weeks ($\bar{X}$ ± SD)	After 4 weeks ($\bar{X}$ ± SD)
Control	10	205.00 ± 35.10	206.00 ± 25.03	224.00 ± 42.48
Model	10	214.00 ± 32.04	225.00 ± 22.61	206.00 ± 21.19
Atorvastatin (10 mg/kg)	10	213.00 ± 56.18	208.50 ± 56.18	208.00 ± 47.56
GANMO (15.1 g/kg)	10	204.00 ± 37.48	218.00 ± 41.04	204.50 ± 48.10
GANMO (45.3 g/kg)	10	222.00 ± 43.92	231.50 ± 53.85	220.00 ± 66.16

**Table 2:** Effect of GANMO on lipid levels in cholesterol-induced dyslipidemia after 2 weeks of treatment.

Groups/treatment 2^nd^ week	*n*	TG ($\bar{X}$ ± SD)	TC ($\bar{X}$ ± SD)	HDL-C ($\bar{X}$ ± SD)	LDL-C ($\bar{X}$ ± SD)
Control group	10	0.50 ± 0.11	2.16 ± 0.15	1.08 ± 0.15	0.85 ± 0.22
Model group	10	0.54 ± 0.17	2.38 ± 0.35	1.16 ± 0.20	1.11 ± 0.19^*∗*^
Atorvastatin (10 mg/kg)	10	0.49 ± 0.12	2.36 ± 0.29	1.06 ± 0.16	1.08 ± 0.34
GANMO (15.1 g/kg)	10	0.74 ± 0.25	2.66 ± 0.28	1.05 ± 0.22	1.27 ± 0.46
GANMO (45.3 g/kg)	10	0.45 ± 0.14	2.56 ± 0.33	0.99 ± 0.17	1.37 ± 0.40

**Table 3:** Effect of GANMO on lipid levels in cholesterol-induced dyslipidemia after 4 weeks of treatment.

Groups/treatment on 4^th^ week	*n*	TG ($\bar{X}$ ± SD)	TC ($\bar{X}$ ± SD)	HDL-C ($\bar{X}$ ± SD)	LDL-C ($\bar{X}$ ± SD)
Control group	10	0.52 ± 0.10	2.06 ± 0.14	0.92 ± 0.25	0.90 ± 0.24
Model group	10	0.83 ± 0.09^*∗∗∗*^	3.55 ± 0.57^*∗∗∗*^	1.38 ± 0.38^*∗∗∗*^	1.79 ± 0.59^*∗∗∗*^
Atorvastatin (10 mg/kg)	10	0.75 ± 0.25	2.93 ± 0.53^Δ^	1.72 ± 0.20^Δ^	0.87 ± 0.27^ΔΔΔ^
GANMO (15.1 g/kg)	10	0.81 ± 0.16	2.82 ± 0.55^ΔΔ^	1.25 ± 0.19	1.20 ± 0.46^Δ^
GANMO (45.3 g/kg)	10	0.91 ± 0.19	3.01 ± 0.39^Δ^	1.45 ± 0.46	1.15 ± 0.38^ΔΔ^

Note: statistical analysis was done with *t* test and ANOVA test, and *p* < 0.05 was considered to be statistically significant; ^*∗∗*, *∗∗∗*^: *p* < 0.01 and *p* < 0.001 compared with control group; ^Δ, ΔΔ, ΔΔΔ^: *p* < 0.05, *p* < 0.01, and *p* < 0.001 compared with the model group.

**Table 4:** Hyperlipidemia model induced by P-407.

Lipid levels (mmol/l)	*n*	Control group ($\bar{X}$ ± SD)	Model group ($\bar{X}$ ± SD)
TG	10	0.93 ± 0.15	6.29 ± 1.24^*∗∗∗*^
TC	10	2.36 ± 0.51	6.91 ± 0.92^*∗∗∗*^
HDL-C	10	1.05 ± 0.23	1.53 ± 0.13^*∗∗∗*^
Non-HDL-C	10	1.31 ± 0.40	5.38 ± 0.88^*∗∗∗*^

Note: ^*∗∗∗*^*p* < 0.001 compared with the control group.

**Table 5:** Effects of GANMO on lipid levels in Poloxamer 407-induced dyslipidemia after 1 week of treatment.

Groups	*n*	Serum lipid levels ($\bar{X}$ ± SD)			
		TG (mmol/L)	TC (mmol/L)	HDL-C (mmol/L)	Non-HDL-C (mmol/L)
Model group	10	6.29 ± 1.24	6.91 ± 0.92	1.53 ± 0.13	5.38 ± 0.88
Atorvastatin (100 mg/kg)	10	6.50 ± 1.92	4.70 ± 1.04^ΔΔΔ^ (↓ 32.0%)	1.63 ± 0.19 (↑ 6.5%)	3.07 ± 1.08^ΔΔΔ^ (↓ 42.9%)
GANMO (30.2 g/kg)	10	4.61 ± 1.52^Δ^ (↓ 26.7%)	5.15 ± 0.68^ΔΔΔ^ (↓ 25.5%)	1.74 ± 0.15^ΔΔ^ (↑ 13.7%)	3.41 ± 0.77^ΔΔΔ^ (↓ 36.6%)
GANMO (90.6 g/kg)	10	3.78 ± 0.72^ΔΔΔ^ (↓ 39.9%)	5.04 ± 1.04^ΔΔΔ^ (↓ 27.1%)	1.71 ± 0.21^Δ^ (↑ 11.8%)	3.33 ± 0.93^ΔΔΔ^ (↓ 38.1%)

Note: statistical analysis was done with *t*-test and ANOVA test, and *p* < 0.05 was considered to be statistically significant; ^*∗∗*, *∗∗∗*^: *p* < 0.01 and *p* < 0.001 compared with the control group; ^Δ, ΔΔ, ΔΔΔ^: *p* < 0.05, *p* < 0.01, and *p* < 0.001 compared with the model group.

## Data Availability

The data used to support the findings of this study are available from the corresponding author upon request.
